# Seroprevalence of SARS-CoV-2 Antibodies among Vaccinated and Non-Vaccinated Adults in the West Bank: Results of a Repeated Cross-Sectional Study

**DOI:** 10.3390/vaccines10081332

**Published:** 2022-08-17

**Authors:** Faisal Awartani, Nouar Mohammad Qutob, Mohammad Rajab Asia

**Affiliations:** Department of Health Sciences, Arab American University of Palestine (AAUP), Ramallah, Palestine

**Keywords:** seroprevalence, SARS-CoV-2, antibodies, asymptomatic, cross sectional, probability sampling

## Abstract

Seroprevalence studies provide an accurate measure of SARS-CoV-2 spread at a population level and the number of undiagnosed individuals. Repeated cross-sectional sero-studies are encouraged to monitor the spread of the virus. The aim of this study is to assess the seroprevalence rate among a random sample of Palestinians residing in the West Bank region of Palestine, especially among those who were not vaccinated and not diagnosed. The study was able to assess the prevalence of asymptomatic cases among the Palestinian adult population. The study also focused on measuring the percentage of adult Palestinians who accepted to get vaccinated across gender and age groups. **Methods:** This second round cross-sectional study involved 1451 participants, who agreed to be interviewed and answer the questionnaire, where 910 of them agreed to participate in the sero-study and donate a blood sample to be tested for antibodies. The sample was randomly selected from the adult population, 18 years or older, living in the West Bank region of Palestine. Serological tests for 910 adequate serum samples were performed using immunoassays for the detection of antibodies against SARS-CoV-2. Sociodemographic information and medical history data were collected. **Results:** Study findings indicate that as of October 2021, there was a seroprevalence rate of 75.9% (30% due to infection with COVID-19 virus and 45.9% due to vaccination) with 95% CI (73.1–78.7). The results indicate that the prevalence of antibodies among those who are unvaccinated and undiagnosed was 45.2% with 95% CI (39.9–50.5%). The average age of participants was 37.6 years old. A total of 49.2% were females, and 50.8% were males. In relation to COVID-19, 13.6% of respondents reported getting infected by COVID-19 with statistically significant difference (*p*-value = 0.001) between males (10.7%) and females (16.5%). In terms of vaccination, 52.8% of respondents reported getting vaccinated with an important difference between males (64.3%) and females (40.9%), (*p*-value < 0.01). **Conclusions:** Our findings reveal a drastic rise in seroprevalence of SARS-CoV-2 antibodies due to infection and vaccination. This information is useful for assessing the degree of herd immunity among the adult population and provides better understanding of the pandemic. Population-based seroprevalence studies should be conducted periodically to monitor the SARS-CoV-2 seroprevalence in Palestine and inform policy makers about the efficacy of the surveillance system and the public compliance with vaccination policies especially among females.

## 1. Introduction

Severe acute respiratory syndrome coronavirus-2 (SARS-CoV-2), a novel virus that causes coronavirus disease 2019 (COVID-19) has infected over 460 million and caused the deaths of over 6 million people worldwide as of 12 March 2022 [1]. However, these figures underestimate the accurate cumulative prevalence or incidence of infection [2] due to the lack of accessibility of diagnostic testing, limited testing capacity [3], and asymptomatic infections [4].

Globally, seroprevalence studies using serologic testing have been recognized to provide better estimates of COVID-19 infection rates and prevalence on a population level in different populations by capturing individuals with mild or no symptoms and others who never underwent diagnostic testing [5]. Assessing the cumulative prevalence is critical to understanding disease transmission rates and for understanding the evolution of the pandemic [5,6]. The true prevalence of infection is believed to be 12.5 times more than the number of PCR-reported infections [7] as there are many asymptomatic infections, which constitute the majority of all SARS-CoV-2 infections yet are less likely to present for diagnostic testing [8,9]. Consequently, the accuracy of such seroprevalence studies has been doubted due to insufficient specificity of the serologic assay for a low prevalence population and due to potential sampling bias [10].

In March 2020 a nationwide lockdown was implemented in Palestine to contain the spread of COVID-19. As of March 2022, approximately 580,165 infections have been recorded, and 5326 people have died in Palestine [11]. Previously, the authors of this study undertook a cross-sectional study [12] which revealed 0% seroprevalence of SARS-CoV-2 among a random sample of adults in the general population in the West Bank region of Palestine. Other studies [13] conducted among visitors to primary health care centers in Palestine showed 36% seroprevalence. A large scale sero survey was conducted by the Palestinian ministry of health and WHO produced a seroprevalence of 40% among the general population who are 10 years or older in the West Bank and Gaza [14]. There is a lack of data on the percentage of undiagnosed Palestinian population with previous mild or asymptomatic COVID-19. This second round cross-sectional study was conducted to assess the seroprevalence of SARS-CoV-2 among a national sample of vaccinated and non-vaccinated adults in the West Bank region of Palestine, be it due to infection with COVID-19 or due to vaccination or both. A substantial seroprevalence of anti-SARS-CoV-2 antibodies in the Palestinian population should provide some measure of protection against future waves of COVID-19 in the country.

## 2. Methods

### 2.1. Study Design and Participants

This second round cross-sectional study was conducted between 14 September 2021 and 21 October 2021. The study included a random sample of adults aged 18 years and above residing in 11 governorates in the West Bank, Palestine. The sample included 1451 individuals randomly selected from households using three-stage cluster sampling. The cluster of households or census tracts is considered to be a geographic location which is comprised of 124 households. The process for conducting cluster sampling included: (1) selection of 125 census tracts (clusters) of households using the sampling frame provided by the Palestinian Central Bureau of Statistics (PCBS), (2) selection of 16 households randomly from each cluster, and (3) selection of individuals at random from the list of individuals who are over the age of 18 and belong to the selected household. The clusters were selected using probability proportional to size (PPS) sampling, see Table 1. The following steps were used to select the number of clusters within each population location: (1) the sampling interval (SI) was calculated as the total number of households (N) divided by the total number of clusters (m) (SI=N/m), (2) a random number R0 between 0 and SI was calculated, and (3) calculated Ri as R0 + i*SI, a cluster is selected in Li if Ri belongs to the interval [Ci-1, Ci].

### 2.2. Data Collection

Sociodemographic information, risk factors, medical history, and COVID-19-related information (e.g., symptoms (due to infection or due to vaccination), vaccination status) were obtained during face-to-face interviews with 1451 participants (Table 2). Upon the interview, 911 participants gave consent to test for antibodies against COVID-19. Blood samples were collected from the participants. Blood samples were centrifuged, and serum was separated, labelled, and stored at −20 °C at the AAUP laboratory until it was used.

Data collection was conducted by healthcare professionals following standardized health protocols (World Health, 2020). Ethics approval was obtained from the Helsinki committee (PHRC/HC/737/20). Participants recruited in the study gave written consent forms prior to participation in the study voluntarily agreeing to participate in the research.

The study was conducted between 14 September 2021 and 21 October. Demographic and health data were collected through face-to-face interviews with the 1451 participants. Blood samples were drawn from 911 participants who agreed to participate in the sero study.

### 2.3. SARS-CoV-2 Antibody Testing

Serological tests for the 911 adequate serum samples were performed using an Elecsys^®^ Anti-SARS-CoV-2 assay by using the Cobas Analyzer cobas e 411 (Roche, Basel, Switzerland). The assay is an immunoassay that uses a recombinant protein representing the nucleocapsid (N) antigen. Serum samples in which the nucleocapsid (N) antigen was not detected were run using Elecsys Anti-SARS-CoV-2 S by using the Cobas Analyzer cobas e 411 (Roche). The assay detects antibodies to SARS-CoV-2 spike protein RBD which are produced by mRNA vaccines. Samples from recovered and vaccinated cases with detected SARS-CoV-2 anti N and SARS-CoV-2 anti S antibodies were run as positive controls.

### 2.4. Statistical Analysis

Both univariate and bivariate inferential statistical methods were used for statistical analysis. Confidence interval using 95% confidence levels are used for estimating the seroprevalence across different segments within the Palestinian adult population living in the West Bank region. The lower and upper confidence limits were calculated. A cross-tabulation method was used to explore bivariate relationships between seroprevalence and gender among other background variables. A chi-square test was utilized for testing statistical significance of bivariate relations among the different variables.

## 3. Results

### Sample Characteristics

In total, 1451 participants were enrolled in the study in which 911 undertook serological testing and blood sampling. Among these, 50.8% were males, 64.4% were married, 27.3% finished preparatory school, and 74.4% were non-refugees. The mean age of participants was 37.6. See Table 2 for detailed sample characteristics.

The main findings of the survey showed that a large majority of adult Palestinians (75.9%) developed antibodies against COVID-19. The results did not show significant difference (*p*-value = 0.23) between males (74.2%) and females (77.5%) in the overall seroprevalence, see Table 3. While we noticed strong difference according to gender when we accounted for the source of antibodies, whether or not it’s due to infection or vaccination. The results showed that the prevalence of antibodies due to infection among females was twice (41.2%) that among males (19.9%),(*p*-value < 0.01). See Table 4. Such result could be attributed to differences in vaccination levels among males and fe-males since the S-protein is detected when the respondent has received an mRNA vaccine type such as Pfizer or Moderna.

The study results showed quite a significant difference (*p*-value < 0.01) in antibody prevalence across age groups in the Palestinian society. The youngest age group (18–29 years) recorded the lowest prevalence of antibodies (69.2%), while the age group (30–50 years) showed a prevalence of 77.9%, while the older group (50 years or older) had the highest prevalence of antibodies (84.1%). Such results could be attributed to the fact that the older population categories were more vaccinated than the younger population categories, see Table 5.

An important factor that explained the variation in levels of antibodies prevalence is the vaccination and infection status among the Palestinian population. A new variable was computed based on the infection status with COVID-19 and vaccination status. The new variable was used to segment the Palestinian population over the age of 18 into four categories described as follows: (a) individuals who became infected and got vaccinated, (b) individuals who became infected and did not get vaccinated, (c) individuals who did not become infected and got vaccinated, and (d) individuals who did not become infected and did not get vaccinated.

**Figure 1:**
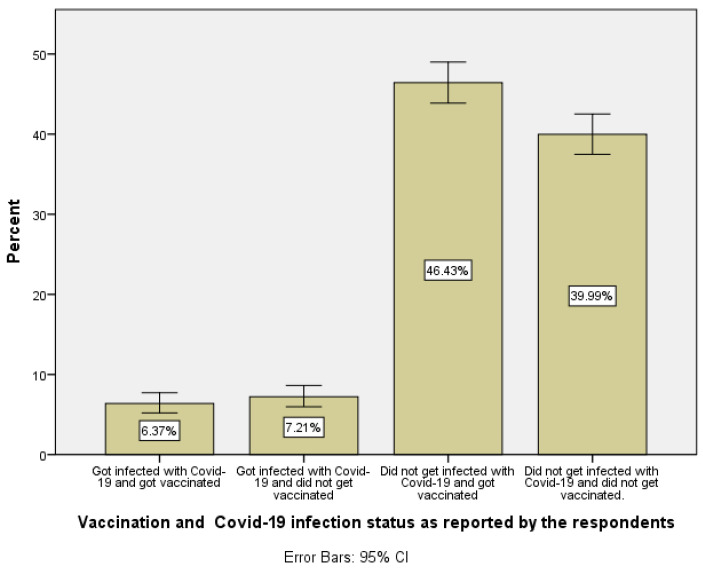
Distribution of vaccination and COVID-19 infection status for adult Palestinians living in the West Bank region of Palestine.

The largest group of individuals within the Palestinian adult population living in the West Bank region are those who did not get infected and got vaccinated (46.4%) followed by the second largest group, which represents those who did not get vaccinated and reported (thought) that they did not get infected (40.0%), see Figure 1.

Our findings showed a strong bivariate relationship between antibody test results and infection–vaccination status (*p*-value < 0.01), see Table 6. It is evident the antibodies prevalence among those who did not become infected and got vaccinated is 94.0% in comparison to 45.2% among those who reported that they did not get vaccinated and did not become infected as well. Interestingly, 45.2% of those who did not get vaccinated and reported that they did not become infected with COVID-19 actually became infected without knowing, concluding this group of the population were asymptomatic to the infection.

The research team at the Arab American University of Palestine conducted a first round of antibody testing against the N-protein on all the 911 blood samples collected from respondents. The second round of antibody tests was conducted against the S-protein among those who received negative results against the N-protein. Of course, those who tested negative against the N-protein and positive against the S-protein are the ones who received the mRNA vaccines.

The data showed a statistically significant difference between males and females in terms of antibody test results due to S-protein availability among those who tested negative for the N-protein (*p*-value < 0.01). See Table 6. Such difference could be attributed to differences in vaccination levels among males and females since the S-protein is detected when the respondent has received an mRNA vaccine type such as Pfizer or Moderna.

When we explored the vaccination patterns among respondents, 52.8% of adult Palestinians residing in the West Bank region reported getting vaccinated, with a significant difference between males (64.3%) and females (40.9%) (*p*-value < 0.01), see Table 7.

When respondents were asked about what type of vaccine they used, 51.8% reported using Pfizer, and 27% reported using Sputnik Light, see Table 8 for more details. There was no significant difference between vaccine type and gender (*p*-value = 0.173).

## 4. Discussion

The present study reports the seroprevalence of anti-SARS-CoV-2 antibodies as of October 2021. There have not been many studies assessing seroprevalence after the beginning of vaccinations. According to this study, seroprevalence of antibodies due to infection with COVID-19 or vaccination one and one-half years after the start of the pandemic and several months after the vaccination rollout was 75.9%, varying among age groups, with the youngest age group having the highest seroprevalence. The high seroprevalence  rate of antibodies observed in our cohort was surely influenced by the high rate of vaccination within the Palestinian adults living in the West Bank region. This finding was similar to a study conducted in Chile which reported a similar seroprevalence of 77.4% five months after initiation of vaccinations [15]. Similar to the Chile study, the results of our study showed a significant difference across gender in antibody prevalence when we account for the source of positivity, whether it is due to infection with COVID-19 or due to vaccination, see Table 9. This result is consistent with other studies which found a statistically significant difference between the prevalence of antibodies and gender [15,16]. At the regional level, a three-phased seroprevalence survey was conducted in Jordan, where results revealed that seroprevalence dramatically increased over time, with only a tiny fraction of seropositive individuals in August 2020 (0.3%), increasing more than 20-fold in October 2020 (7.0%) and reaching one-third of the overall population exposed by the end of 2020 (34.2%) [16]. Such results are compatible with similar seroprevalence surveys conducted by the Palestinian ministry of health and WHO at the end of 2020 [14], while similar results were obtained by a seroprevalence survey conducted by the Arab American University during June 2020 [12]. The most recent serology survey conducted by the CDC from September 2021 until December 2021 in the US showed that seroprevalence increased from 36.5% (95% CI = 35.7–37.4) to 63.7% (95% CI = 62.5–64.8) among adults aged 18–49 years, 28.8% (95% CI = 27.9–29.8) to 49.8% (95% CI = 48.5–51.3) among those aged 50–64 years, and from 19.1% (95% CI = 18.4–19.8) to 33.2% (95% CI = 32.2–34.3) among those aged ≥65 years [17]. Approximately similar seroprevalence due to infection with COVID-19 (30%) was obtained by our serology survey that targeted adults over the age of 18 during September 2021, see Table 9.

Two years after the start of the COVID-19 pandemic, the actual prevalence of infection is still underestimated compared with PCR-confirmed COVID-19 cases. Older individuals have lower antibody levels after vaccination compared with younger individuals. Approximately 40% of the Palestinian adult population over the age of 18 thought they were not infected and did not make the effort to get vaccinated. The study’s findings showed 45% of those who thought they were not infected and did not get vaccinated, tested positive for SARS-CoV-2 antibodies. By multiplying these two percentages, one can conclude that 18% of the Palestinian adult population who are over the age 18 got infected with SARS-CoV-2 without knowing.

## 5. Conclusions

Our findings reveal a drastic rise in seroprevalence of SARS-CoV-2 antibodies due to infection and vaccination. This information is useful for assessing the degree of herd immunity among the adult population and provides better understanding of the pandemic. Population-based seroprevalence studies should be conducted periodically to monitor the SARS-CoV-2 seroprevalence in Palestine and inform policy makers about the efficacy of the surveillance system and the public compliance with vaccination policies, especially among females.

Special attention should be given to estimating the size of the population segment who got infected with COVID-19 without knowing, that is those who were asymptomatic. This group of people could have contributed to a faster transmission rate in the spread of the virus, since they had no symptoms. Governments and public health officials should put more effort in detecting asymptomatic cases through conducting seroprevalence surveys on a regular basis and developing regulations to encourage more people to get vaccinated. Tracking seroprevalence due to vaccination is an important issue, since vaccination contributes to the reduction of hospitalization among COVID-19 patients. Preliminary evidence has highlighted a possible association between severe COVID-19 and persistent cognitive deficits [18]. Further research is required to confirm this association, determine whether cognitive deficits relate to clinical features from the acute phase or to mental health status at the point of assessment, and quantify the rate of recovery.

## Figures and Tables

**Table 1:** The PPS sampling algorithm for selecting population locations.

Location	Number of Households in each cluster(census tract)	Cumulative
L1	X1	C1 = X1
L2	X2	C2 = X1 + X2
L3	X3	C3 = X1 + X2 + X3
Lk-1	Xk-1	Ck-1 = X1 + X2 + … + Xk-1
Lk	Xk	Ck = X1 + X2 + … + Xk

PPS, probability proportional to size.

**Table 2:** Sample characteristics.

Background Variable		N	%
Gender	Male	737	50.8%
	Female	714	49.2%
Marital Status	Never married	377	26.0%
	Engaged	33	2.3%
	Married	935	64.4%
	Divorced	21	1.4%
	Widowed	81	5.6%
	Separated	5	0.3%
Highest Degree Obtained	Illiterate	57	3.9%
	Knows how to read and write	68	4.7%
	Elementary	216	14.9%
	Preparatory	396	27.3%
	Secondary	336	23.2%
	Two or one-year diploma	81	5.6%
	Bachelor	273	18.8%
	Higher diploma	2	0.2%
	Masters	18	1.3%
	Ph.D.	3	0.2%
Refugee Status	Registered refugee	357	24.6%
	Unregistered refugee	15	1.0%
	Not a refugee	1078	74.3%
Age category in years	18–29	561	38.6%
	30–49	568	39.2%
	50+	322	22.2%

**Table 3:** Antibodies test results irrespective of the source (infection or vaccine) by gender.

			Gender		Total
			Male	Female	
Antibodies test result	Negative	Count	114	105	219
		% within Gender	25.8%	22.4%	24.1%
	Positive	Count	328	363	691
		% within Gender	74.2%	77.6%	75.9%
Total		Count	442	468	910
		% within Gender	100.0%	100.0%	100.0%
**Chi-Square Tests**					
	Value	Df	Asymptotic Significance (2-sided)	Exact Sig. (2-sided)	Exact Sig. (1-sided)
Pearson Chi-Square	1.401 ^a^	1	0.237		
Continuity Correction ^b^	1.223	1	0.269		
Likelihood Ratio	1.401	1	0.237		
Fisher’s Exact Test				0.245	0.134
Linear-by-Linear Association	1.399	1	0.237		
N of Valid Cases	910				

^a^ 0 cells (0.0%) have expected count less than 5; the minimum expected count is 106.37; ^b^ computed only for a 2 × 2 table.

**Table 4:** Antibody test results by Protein Type and Gender.

Antibodies Test Results by Protein Type and Gender Crosstabulation					
			Gender		Total
			Male	Female	
Antibody Test Results	Negative	Count	114	105	219
		% within Gender	25.8%	22.4%	24.1%
	N-Protein Positive	Count	186	289	475
		% within Gender	42.1%	61.8%	52.2%
	S-Protein Positive and N-protein Negative	Count	142	74	216
		% within Gender	32.1%	15.8%	23.7%
Total		Count	442	468	910
		% within Gender	100.0%	100.0%	100.0%
**Chi-Square Tests**					
	Value	df		Asymptotic Significance (2-sided)	
Pearson Chi-Square	43.405 ^a^	2		0.000	
Likelihood Ratio	43.916	2		0.000	
Linear-by-Linear Association	0.345	1		0.557	
N of Valid Cases	910				

^a^ 0 cells (0.0%) have expected count less than 5; the minimum expected count is 104.91.

**Table 5:** Antibodies test results by age category.

			Age Category in Years			Total
			18–29	30–49	50+	
Antibodies test result	Negative	Count	113	71	35	219
		% within Age category in years	30.8%	22.0%	15.8%	24.1%
	Positive	Count	254	251	186	691
		% within Age category in years	69.2%	78.0%	84.2%	75.9%
Total		Count	367	322	221	910
		% within Age category in years	100.0%	100.0%	100.0%	100.0%
**Chi-Square Tests**						
		Value	df	Asymptotic Significance (2-sided)		
Pearson Chi-Square		17.986 ^a^	2	0.000		
Likelihood Ratio		18.297	2	0.000		
Linear-by-Linear Association		17.789	1	0.000		
N of Valid Cases		910				

^a^ 0 cells (0.0%) have expected count less than 5; the minimum expected count is 53.19.

**Table 6:** Antibodies test results by vaccination and COVID-19 infection status as reported by respondents.

			Antibodies Test Result		Total
			Negative	Positive	
Vaccination and COVID-19 infection status as reported by the respondents	Became infected with COVID-19 and got vaccinated	Count	1	59	60
			1.7%	98.3%	100.0%
	Became infected with COVID-19 and did not get vaccinated	Count	4	67	71
			5.6%	94.4%	100.0%
	Did not become infected with COVID-19 and got vaccinated	Count	26	410	436
			6.0%	94.0%	100.0%
	Did not become infected with COVID-19 and did not get vaccinated.	Count	188	155	343
			54.8%	45.2%	100.0%
Total		Count	219	691	910
			24.1%	75.9%	100.0%
**Chi-Square Tests**					
	Value	df	Asymptotic Significance (2-sided)		
Pearson Chi-Square	285.275 ^a^	3	0.000		
Likelihood Ratio	294.040	3	0.000		
Linear-by-Linear Association	62.356	1	0.000		
N of Valid Cases	910				

^a^ 0 cells (0.0%) have expected count less than 5; the minimum expected count is 14.44.

**Table 7:** Vaccination status as reported by respondents by gender.

Crosstab					
			Gender		Total
			Male	Female	
Have you been vaccinated against COVID-19?	Yes	Count	474	292	766
		% within Gender	64.3%	40.9%	52.8%
	No	Count	263	422	685
		% within Gender	35.7%	59.1%	47.2%
Total		Count	737	714	1451
		% within Gender	100.0%	100.0%	100.0%
**Chi-Square Tests**					
	Value	Df	Asymptotic Significance (2-sided)	Exact Sig. (2-sided)	Exact Sig. (1-sided)
Pearson Chi-Square	79.805 ^a^	1	0.000		
Continuity Correction ^b^	78.868	1	0.000		
Likelihood Ratio	80.540	1	0.000		
Fisher’s Exact Test				0.000	0.000
Linear-by-Linear Association	79.750	1	0.000		
N of Valid Cases	1451				

^a^ 0 cells (0.0%) have expected count less than 5; the minimum expected count is 337.07; ^b^ computed only for a 2 × 2 table.

**Table 8:** Type of vaccine used by gender.

Crosstab					
			Gender		Total
			Male	Female	
Name of vaccine	Pfizer	Count	244	153	397
		% within Gender	51.4%	52.4%	51.8%
	Sputnik	Count	12	7	19
		% within Gender	2.5%	2.4%	2.5%
	AstraZeneca	Count	28	18	46
		% within Gender	5.9%	6.2%	6.0%
	Sputnik Light	Count	120	87	207
		% within Gender	25.3%	29.8%	27.0%
	Moderna	Count	44	14	58
		% within Gender	9.3%	4.8%	7.6%
	Sinopharm	Count	19	12	31
		% within Gender	4.0%	4.1%	4.0%
	Others	Count	8	1	9
		% within Gender	1.7%	0.3%	1.2%
Total		Count	475	292	767
		% within Gender	100.0%	100.0%	100.0%
**Chi-Square Tests**					
		Value	df	Asymptotic Significance (2-sided)	
Pearson Chi-Square		9.002 ^a^	6	0.173	
Likelihood Ratio		9.853	6	0.131	
Linear-by-Linear Association		0.779	1	0.377	
N of Valid Cases		767			

^a^ 1 cells (7.1%) have expected count less than 5. The minimum expected count is 3.43.

**Table 9:** Seroprevalence according to gender and source of antibodies (infection or vaccine).

Antibody Test Result by Gender Crosstabulation					
			Gender		Total
			Male	Female	
Antibody Test Result	Negative	Count	114	105	219
		% within Gender	25.7%	22.4%	24.0%
	positive due to infection with COVID-19	Count	88	193	281
		% within Gender	19.9%	41.2%	30.8%
	positive due to vaccination	Count	241	170	411
		% within Gender	54.4%	36.3%	45.1%
Total		Count	443	468	911
		% within Gender	100.0%	100.0%	100.0%
**Chi-Square Tests**					
		Value	df	Asymptotic Significance (2-sided)	
Pearson Chi-Square		51.222 ^a^	2	0.000	
Likelihood Ratio		52.214	2	0.000	
Linear-by-Linear Association		7.673	1	0.006	
N of Valid Cases		911			

^a^ 0 cells (0.0%) have expected count less than 5; the minimum expected count is 106.50.

## Data Availability

Data set used in this research can be accessed by direct contact with the authors or department of health sciences at AAUP the owner of the data.
